# Oral exposure to PET microplastics induces the pancreatic immune response and oxidative stress in immature pigs

**DOI:** 10.1186/s12864-025-11760-1

**Published:** 2025-07-01

**Authors:** Karol Mierzejewski, Aleksandra Kurzyńska, Monika Golubska, Ismena Gałęcka, Jarosław Całka, Iwona Bogacka

**Affiliations:** 1 Department of Animal Anatomy and Physiology, University of Warmia and Mazury in Olsztyn, Oczapowskiego 1a, Olsztyn, 10-719 Poland; 2 Department of Epizootiology, University of Warmia and Mazury in Olsztyn, Oczapowskiego 14, Olsztyn, 10-719 Poland; 3 Department of Clinical Physiology, University of Warmia and Mazury in Olsztyn, Oczapowskiego 14, Olsztyn, 10-719 Poland

**Keywords:** Diabetes, Pig, Pancreas, Environmental hazard, Cytokines, Chemokines, Risk factor

## Abstract

Microplastics are a widespread environmental hazard and their impact on human health has become a growing concern in last years. Recently, the potential role of microplastics in the development of various diseases, including diabetes, has been highlighted. Therefore, the aim of this study was to investigate the effects of PET microplastics on the pancreas using immature pigs as a model organism. We analyzed the global transcriptomic profile of the pancreas by RNA-Seq in piglets treated with either a low (0.1 g/day) or a high dose (1 g/day) of PET microplastics for 4 weeks. The analysis revealed a dose-dependent effect of PET microplastics on gene expression. A low dose changed the expression of one gene, while a high dose affected the expression of 86 genes. The differentially expressed genes, including immune cell markers, cytokines and chemokines, may activate the immune system in the pancreas in a way that is characteristic of the pathogenesis of diabetes. In addition, PET microplastics induced oxidative stress in the pancreas. These above imply that oral exposure to PET microplastics could be a new risk factor for the development of diabetes.

**Supplementary Information:**

The online version contains supplementary material available at 10.1186/s12864-025-11760-1.

## Introduction

Microplastics (MPs) are plastic particles smaller than 5 mm, which can be either produced intentionally (primary) or generated by the degradation of larger plastic parts (secondary) [1]. Their ubiquitous presence in water, food, and air has raised great concern about their impact on human health in recent years. Recent studies have confirmed the presence of MPs in human tissues including the placenta, testicles, liver, brain and blood [2–4]. Furthermore, investigations have demonstrated that microplastic particles can influence various physiological processes, such as activation of the immune response, triggering inflammation and inducing oxidative stress [5–7]. There is evidence that MPs may contribute to metabolic diseases associated with insulin resistance by dysregulating the gut microbiota [8] transferring harmful microorganisms and metabolites to the liver via the gut-liver axis [9] or affecting blood glucose levels [10]. In addition, we have previously shown that PET microplastics alter the expression of miRNA in serum-derived extracellular vesicles (EVs), which are associated with insulin resistance and the development of type II diabetes [11].

Diabetes mellitus (DM) is a long-term metabolic disease characterized by constantly elevated blood glucose levels, either due to insufficient insulin production or the body’s inability to effectively use the insulin it produces [12]. This disease affects people of all ages, genders, and regions, making it one of the leading causes of mortality and morbidity worldwide. Therefore, diabetes is considered one of the fastest growing health challenges of the twenty-first century and represents a significant financial burden for healthcare systems worldwide. An estimated 10.5% of the world’s population currently live with this disease [13]. Based on their pathogenesis, two main types of diabetes can be distinguished: type 1 diabetes (T1D) and type 2 diabetes (T2D) [14].

Dysregulation of insulin synthesis is closely related to the pathophysiology of both T1D and T2D [15, 16]. Insulin is known to be a central player in the regulation of metabolism, particularly in the maintenance of glucose homeostasis [17]. There is also evidence that dysregulation of insulin signaling is linked to the development of cognitive impairment and dementia [18]. Studies in animal models have shown that insulin resistance and impaired insulin signaling exacerbate neurodegenerative processes in Alzheimer’s disease by affecting amyloid-beta accumulation and neuronal deficits [19, 20]. Therefore, understanding the mechanisms that impair insulin synthesis is essential to describe the processes underlying many diseases.

In light of our previous findings and the existing literature, we conducted this study to determine the impact of PET microplastics on the global transcriptomic profile and oxidative status of the pancreas, using immature piglets as a model organism. The pig is considered a valuable model for the study of diabetes due to its physiological similarities to humans, particularly in terms of pancreatic structure, metabolic function, and pathophysiological responses [21]. These similarities make pigs particularly relevant for investigating complex diseases such as diabetes and metabolic syndrome, which involve insulin resistance. Moreover, the metabolic pathways involved in insulin and glucose regulation in pigs are comparable to those in humans. In this study, we used two doses of PET microplastics – a low dose of 0.1 g/day/animal and a high dose of 1 g/day/animal. The doses were selected on the basis of estimates that humans consume an average of 0.1 to 5 g of microplastics per week [22]. However, determining the precise intake of microplastics is a challenge as exposure levels varies depending on individual lifestyle and environmental factors. Nevertheless, the doses used in this study – approximately 0.7 and 7 g per week – appear to reflect a realistic level of human exposure based on average intake estimates. Furthermore, it is important to emphasize that the average exposure of infants to PET is significantly higher than that of adults [23], and the main objective of our study was to investigate the effects on a young organism.

## Results

### The effect of PET microplastics on differential gene expression in pancreas

**Fig. 1:**
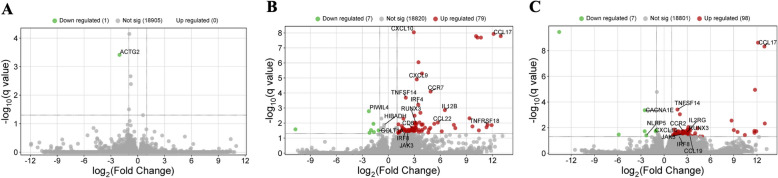
Volcano plots illustrating changes in gene expression profiles in porcine pancreas under the influence of PET microplastics. Plot **A** shows gene expression changes following a low dose of PET microplastics treatment, plot **B** shows changes after a high dose, and plot **C** compares gene expression between high and low doses of PET microplastics. The X-axis displays the logarithmic fold changes in expression (log2 FC), while the Y-axis represents the -log10 transformed adjusted *p*-values (q-values). A horizontal dashed line marks the significance threshold for the adjusted *p*-value (0.05), and vertical dashed lines denote the fold change cut-off (absolute value of log2 FC > 1.5). Each dot represents a gene’s expression level, with red dots indicating significantly overexpressed genes and green dots indicating significantly underexpressed genes (both with an adjusted *p*-value < 0.05). Grey dots represent genes with non-significant changes in expression

**Fig. 2:**
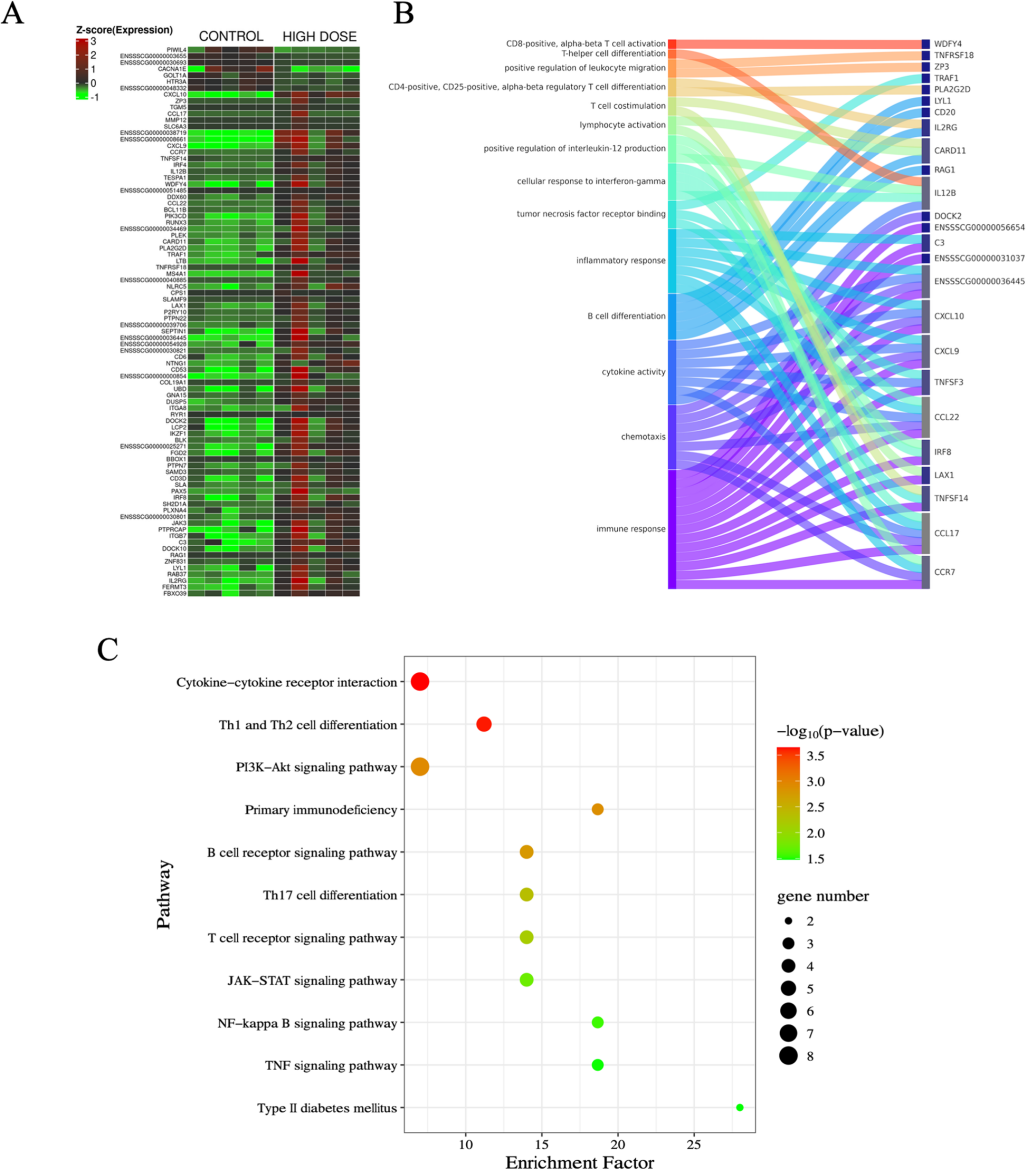
Gene expression analysis in porcine pancreas after exposure to high dose of PET microplastics. **A** Heatmap showing the expression levels of differentially expressed genes in control and high dose PET microplastics-treated groups. Rows represent individual genes, and columns represent samples from each treatment group (control vs. high dose). Colors indicate the level of gene expression, with red representing upregulated genes, green representing downregulated genes, and black indicating no significant change in expression. The dendrogram on the left clusters genes based on similarity in expression patterns. **B** Sankey diagram illustrating enriched biological processes and pathways associated with the differentially expressed genes in the high dose of PET microplastics-treated group. This diagram shows how specific genes (on the right) are linked to various biological processes (on the left). Each pathway is represented by a different color, and lines connect genes to the biological processes in which they are involved. **C** Bubble plot showing pathway enrichment analysis for the genes affected by high dose of PET microplastics. Each pathway is listed on the Y-axis, and the X-axis displays the rich factor, which represents the ratio of differentially expressed genes associated with each pathway to the total number of genes in that pathway. The color of each bubble indicates the statistical significance (*p*-value) of the enrichment, with a gradient from red (less significant) to blue (more significant). The size of the bubbles represents the number of genes associated with each pathway

Treatment of piglets with a low dose of PET microplastics revealed only one differentially expressed gene (DEG) in the pancreas – actin gamma 2 (*ACTG2*), which was downregulated (Fig. 1A). In contrast, treatment with a high dose of PET microplastics resulted in the identification of 86 DEGs, with 7 genes being downregulated and 79 upregulated (Figs. 1B, 2A). Gene Ontology (GO) annotation of biological processes (BP) included 533 terms, while 174 terms were classified under molecular functions (MF) and 94 under cellular components (CC). According to GO analysis, the DEGs involved in the pancreatic response to PET microplastics were associated with processes such as immune response (C–C motif chemokine receptor 7 (*CCR7*), C–C motif chemokine ligand 17 (*CCL17*), tumor necrosis factor superfamily member 14 (*TNFSF14*), lymphocyte activation family member X1 (*LAX1*), interferon regulatory factor 8 (*IRF8*), C–C motif chemokine ligand 22 (*CCL22*), C-X-C motif chemokine ligand 9 (*CXCL9*), C-X-C motif chemokine ligand 10 (*CXCL10*), lymphotoxin beta (*LTB*), complement C3 (*C3*), *ENSSSCG00000056654*, *ENSSSCG00000036445*)), chemotaxis (*CCR7, CCL17, CCL22, CXCL9, CXCL10, ENSSSCG00000036445*, dedicator of cytokinesis 2 (*DOCK2*)), cytokine activity (interleukin 12B (*IL12B*), *CCL17, CCL22, LTB, CXCL9, CXCL10, ENSSSCG00000036445*), and B cell differentiation (recombination activating 1 (*RAG1*), caspase recruitment domain family member 11 (*CARD11*), interleukin 2 receptor subunit gamma (*IL2RG*), membrane spanning 4-domains A1 (*MS4 A1*), lymphoblastic leukemia associated hematopoiesis regulator 1 (LYL1)) (Fig. 2B). KEGG pathway analysis indicated that the identified DEGs were involved in cytokine − cytokine receptor interaction (*IL12B, CCR7, CCL17, TNFSF14*, tumor necrosis factor receptor superfamily member 18 (*TNFRSF18*), *CCL22, LTB, CXCL9, CXCL10, ENSSSCG00000036445, IL2RG*), Th1 and Th2 cell differentiation (*IL12B*, runt-related transcription factor 3 (*RUNX3*), CD3 delta subunit (*CD3D*), *IL2RG*, Janus kinase 3 (*JAK3*)), T cell receptor signaling pathway (*CARD11*, phosphoinositide-3-kinase catalytic subunit delta (*PIK3 CD*), *CD3D*, lymphocyte cytosolic protein 2 (*LCP2*)), Toll-like receptor signaling pathway (*IL12B, PIK3 CD, CXCL9, CXCL10*), NF-kappa B signaling pathway (*TNFSF14, CARD11, LTB,* TNF receptor associated factor 1 (*TRAF1*)), and the JAK-STAT signaling pathway (*IL12B, PIK3 CD, IL2RG, JAK3*) (Fig. 2C). The list of the top 20 DEGs, along with detailed results of the DEGs, GO and KEGG analyses can be found in Tables S1, S2, S3, S4.

### Comparison between the low and high doses of PET microplastics

**Fig. 3:**
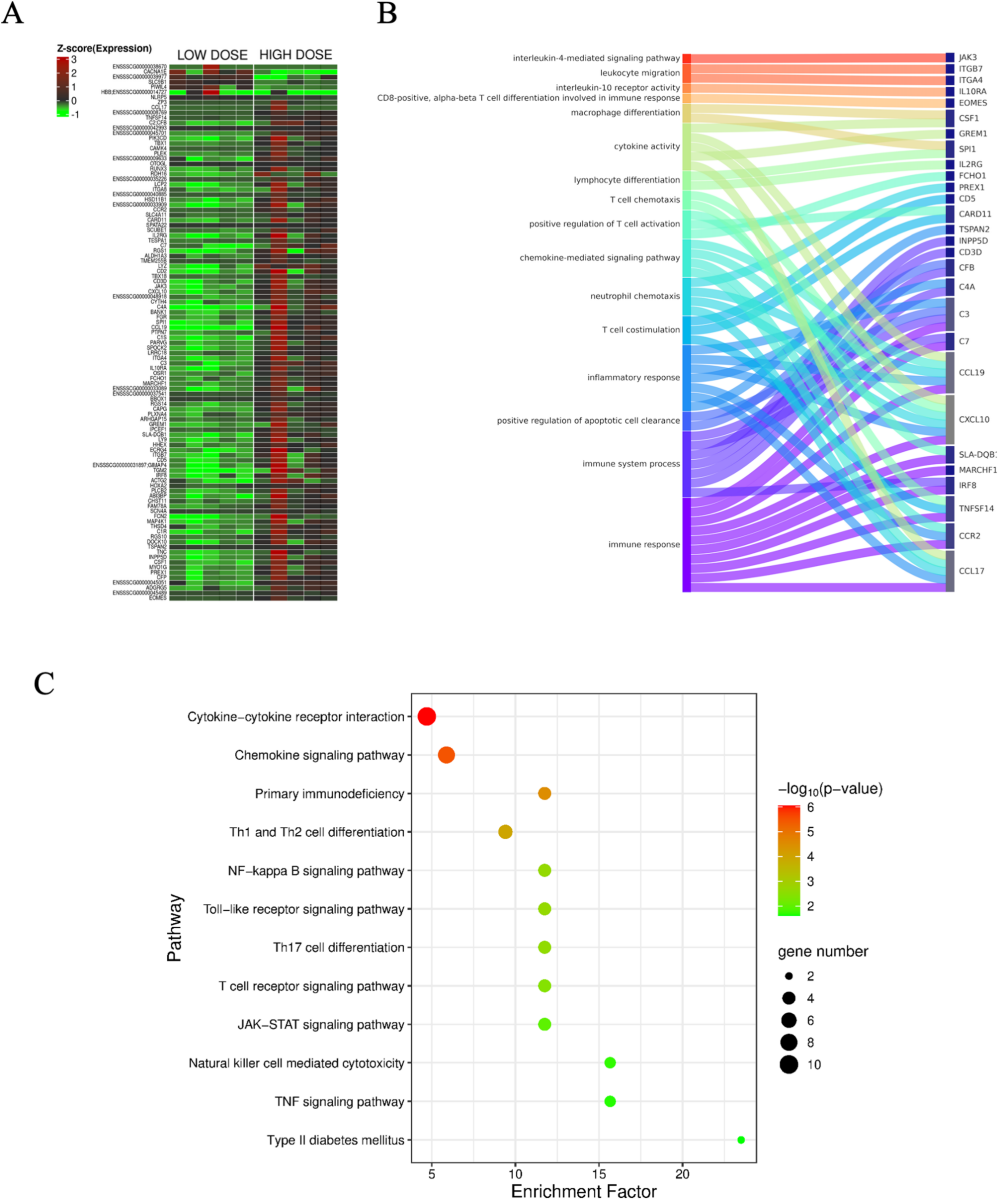
Gene expression analysis in porcine pancreas comparing the effects of high and low doses of PET microplastics. **A** Heatmap displaying the expression levels of differentially expressed genes in the pancreas for both high dose and low dose PET microplastics treatment groups. Rows represent individual genes, and columns represent samples from each treatment group (low dose vs. high dose). Colors indicate the level of gene expression, with red representing upregulated genes, green indicating downregulated genes, and black showing genes with no significant change in expression. The dendrogram on the left clusters genes based on similarity in their expression patterns across both doses. **B** Sankey diagram illustrating enriched biological processes and pathways linked to the differentially expressed genes when comparing high and low doses of PET microplastics. This diagram shows how specific genes (on the right) connect to biological processes (on the left), such. Each pathway is represented by a different color, with lines connecting genes to the biological processes they influence, highlighting differences in pathway involvement between the two doses. **C** Bubble plot representing pathway enrichment analysis for genes with differential expression between high dose and low dose PET microplastics exposure. Pathways are listed on the Y-axis, while the X-axis shows the "rich factor, "which reflects the ratio of differentially expressed genes in each pathway to the total number of genes in that pathway. The color gradient of each bubble indicates the statistical significance (p-value) of the enrichment, with a range from red (less significant) to blue (more significant). The size of the bubbles represents the number of genes associated with each pathway

The analysis identified 105 differentially expressed genes (DEGs) in the pancreas of gilts administered a high dose of PET microplastics (MPs), compared to those given a low dose. Of these, 7 genes were downregulated and 98 were upregulated (Figs. 1C, 3A). The Gene Ontology (GO) annotation of biological processes (BP) included 663 terms, while 195 terms were classified under molecular functions (MF) and 105 under cellular components (CC). Most of the processes were associated with immune system activation, including immune response (*CCL17*, C–C motif chemokine receptor 2 (*CCR2*), *TNFSF14*, *IRF8*, membrane associated ring-CH-type finger 1 (*MARCHF1*), swine leukocyte antigen-DQB1 (*SLA-DQB1*), *CXCL10,* C–C motif chemokine ligand 19 (*CCL19*), complement C7 (*C7*), *C3*), follicular B cell differentiation (*IRF8*, SPI-1 proto-oncogene (*SPI1*)), inflammatory response (tetraspanin 2 (*TSPAN2*), *CCL17*, CCR2, complement C4 A (*C4 A*), *CXCL10, CCL19, C3*), and T cell costimulation (*TNFSF14, CARD11*, CD5 molecule (*CD5*)) (Fig. 3B). KEGG pathway analysis revealed that the DEGs were involved in the chemokine signaling pathway (FGR proto-oncogene, SRC family tyrosine kinase (FGR), phospholipase C beta 2 (*PLCB2*), *CCL17, CCR2, PIK3 CD, CXCL10, CCL19*, phosphatidylinositol-3,4,5-trisphosphate-dependent RAC exchange factor 1 (*PREX1*), *JAK3*), cytokine − cytokine receptor interaction (*CCL17, CCR2, TNFSF14, CXCL10*, interleukin 10 receptor subunit alpha (*IL10RA*), *CCL19, IL2RG, ENSSSCG00000009633*, colony stimulating factor 1 (*CSF1*)), Th1 and Th2 cell differentiation (*RUNX3, SLA-DQB1, CD3D, IL2RG, JAK3*), and the PI3 K − Akt signaling pathway (*PIK3 CD*, tenascin C (*TNC*), integrin subunit alpha 8 (*ITGA8*), *IL2RG*, integrin subunit alpha 4 (*ITGA4*), *JAK3*, *CSF1*, integrin subunit beta 7 (*ITGB7*)) (Fig. 3C). The list of the top 20 DEGs, along with detailed results of the DEGs, GO and KEGG analyses can be found in Tables S1, S2, S3, S4.

### Real-time PCR

The analysis revealed that the mRNA abundance of CCL22, CXCL10, CXCL9, IL12B, IL2R, TNFSF14, IRF8, JAK3, was upregulated in the pancreas of piglets treated with a high dose of PET microplastics. Notably, the expression patterns observed for these DEGs were consisted with results obtained from RNA-Seq analysis (Supplemental Fig. 1).

### Oxidative stress markers

**Fig. 4:**
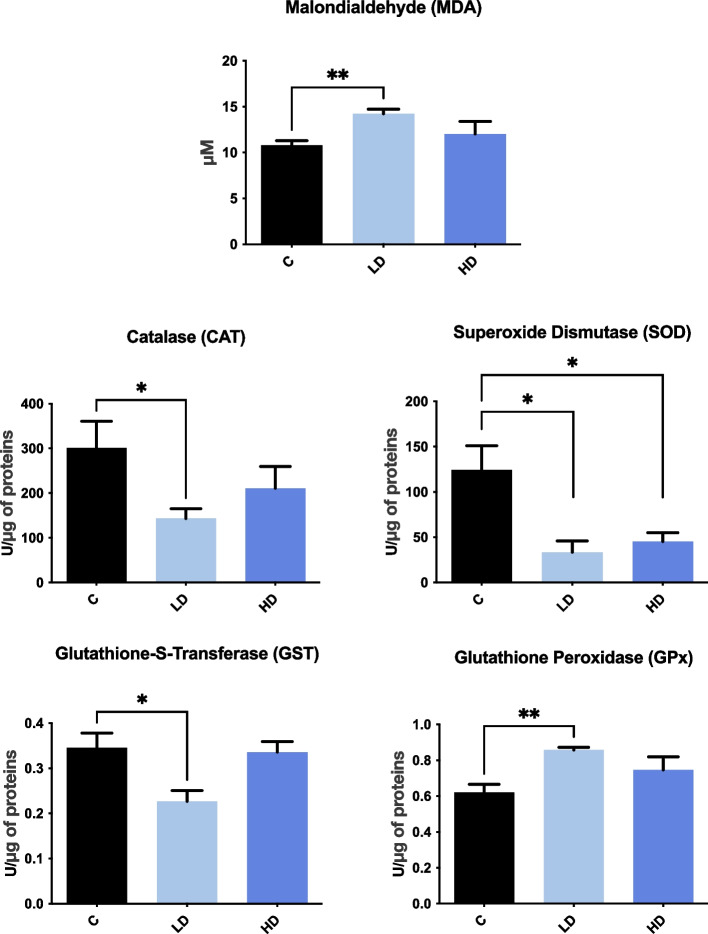
Activity level of oxidative stress marker (MDA) and antioxidant enzymes (GPx, SOD, CAT, GST) in the pancreas of the experimental groups that received a low dose (LD) and a high dose (HD) of PET microplastics compared to the control (C). Significant differences between the groups are indicated by asterisks (*p < 0.05, **p < 0.01)

Exposure of piglets to a low dose of PET microplastics led to an increase in pancreatic malondialdehyde (MDA) levels, an indicator of lipid peroxidation. Moreover, the low dose of PET microplastics reduced the activity of the following antioxidants in the pancreas: catalase (CAT), superoxide dismutase (SOD), and glutathione-S-transferase (GST), while it increased glutathione peroxidase (GPx) activity. In contrast, significant changes following treatment with a high dose of PET microplastics were observed only in SOD activity, which was decreased (Fig. 4).

## Discussion

This study provides evidence that PET microplastics may pose a potential threat by altering pancreatic physiology. We demonstrated that PET microplastics alter the transcriptome profile and markers of oxidative stress in the pancreas. Notably, the differentially regulated genes, particularly at the high dose, are involved in the regulation of cytokine and chemokine activity, lymphocyte differentiation, TLR4 and NF-kappa B signaling pathways. Additionally, our findings indicate that PET microplastics increased the expression of *IL-12β* in the pancreas, a cytokine previously established as crucial role in the development and pathogenesis of autoimmune diseases by driving the recruitment of inflammatory cells [24]. In non-obese diabetic (NOD) mice, increased expression of this cytokine was found in islet cells, in parallel with the destruction of β-cells [25]. Another study in NOD mice indicated that IL-12 plays a significant role in the transition from non-destructive to destructive insulitis [26, 27]. Elevated levels of IL-12 were also associated with increased insulin resistance and impaired glucose tolerance [28].

Our study showed that PET microplastics upregulated the expression of various members of the TNF superfamily such as *TNFSF3* (LTβ), *TNFSF14* (LIGHT) and *TNFRSF18* (GITRL). These pro-inflammatory cytokines affect both the immune system and β-cell function by binding to their corresponding receptors (TNFRSF) [29]. Evidence suggests that TNFSF3 receptors, which are predominantly expressed on immune cells and lymphoid stromal cells, contribute to the ectopic formation of tertiary lymphoid organs (TLOs), which trigger chronic inflammation and autoimmune responses [30]. Such processes facilitate persistent immune activation and β-cell destruction, thereby promoting the development and progression of type 1 diabetes [31, 32]. Additionally, TNFSF14 has been linked to immune-mediated destruction of β-cells in diabetic mice [33], and elevated levels of this cytokine have been observed in patients with type 2 diabetes [34]. TNFRSF18, in turn, exacerbates autoimmune diabetes by selectively activating aggressive T cells while suppressing Tregs. It has been shown that blocking TNFRSF18 expression can prevent the onset of diabetes [35]. Collectively, these findings suggest that PET microplastics may increase the risk of β-cell damage and contribute to the development of diabetes by upregulating key cytokines such as *IL-12β*, *TNFSF3*, *TNFSF14* and *TNFRSF18*.

Exposure to a high dose of PET microplastics was found to increase the expression of several chemokines in the pancreas, including *CXCL10*, *CXCL9*, *CCL17* and *CCL22*. Chemokines are specific cytokines that mediate the chemotaxis of immune cells by binding to surface receptors. Elevated levels of CXCL10 have been found in the serum of patients with newly diagnosed type 1 or type 2 diabetes and in patients at high risk of developing the disease [36–39]. Further studies conducted in mouse models have shown that CXCL10 is overexpressed in the pancreatic islets even prior detectable onset of insulitis [40, 41]. Moreover, CXCL9, in conjunction with CXCL10, also changes the mass and function of β-cells, and deletion of CXCR3 in mice delays the onset of type 1 diabetes [42]. Thus, PET microplastics may activate immune response in the pancreas and increase the risk of type 1 diabetes. Interestingly, we also observed an increased expression of *CCL17* and *CCL22*, which act via the CCR4 receptor, which is predominantly localized on Tregs. These chemokines are known to protect pancreatic β-cells from autoimmune attack in type 1 diabetes. CCL17 and CCL22 recruit Tregs to the islets, reducing autoreactive CD8 + T cells and mitigating β-cell destruction [43]. The simultaneous upregulation of chemokines that promote (*CXCL10, CXCL9*) and protect against (*CCL17, CCL22*) type 1 diabetes suggests a complex immune response to PET microplastics. Initially, microplastics may promote β-cell destruction, but tissue injury could trigger a feedback mechanism that produces protective chemokines, such as CCL17 and CCL22, to limit further damage.

The current research provides evidence that PET microplastics may modify the immune response by upregulating the expression of markers associated with T cell infiltration such as *CD3D*, *CD6, RUNX3* and *LCP2* [44, 45]. Furthermore, PET microplastics upregulated the expression of *JAK3*, a key enzyme in the JAK-STAT signaling pathway involved in regulating immune cell activity. JAK3, in combination with the IL-2 receptor, which was also upregulated in this study, plays a critical role in T cell development and the progression of type 1 diabetes [46]. Inhibition of JAK3 has been shown to protect against diabetes onset in mouse models, highlighting its importance in diabetes pathogenesis [47]. Though T cells are largely responsible for β-cell destruction in type 1 diabetes, there is growing evidence that B cells also play a role in the pathogenesis of the disease. Notably, there is evidence that depleting B cells delays the progression of type 1 diabetes in newly diagnosed patients [48]. In our study, PET microplastics increased the expression of markers important for B cell activation [49] such as *CD20* (MS4 A1), *PAX5* and *BLK*, what suggest that PET microplastics may accelerate autoimmune processes leading to β-cell dysfunction. The upregulation of both T cell and B cell markers following exposure to PET microplastics implicates these particles in immune dysregulation associated with type 1 diabetes.

Consistent with these immune-related changes, the present study showed an increased expression of interferon regulatory factors (IRFs), particularly *IRF4* and *IRF8*. It is worth noting that both factors play a crucial role in the pathogenesis of type 1 diabetes by modulating interactions between β-cells and immune cells [32]. Overactivation of IRF4 has been associated with increased secretion of diabetogenic cytokines by CD4 + T cells, promoting the development of type 1 diabetes [50, 51]. IRF8, in turn, is essential for the maturation of dendritic cells (DCs) and the production of type I interferons, which may exacerbate autoimmune attacks on β-cells [32, 52].

In addition to transcriptomic effects, the colorimetric analysis showed that a low dose of PET microplastics had a stronger effect on the oxidative status of the pancreas than a high dose. Oxidative stress can be assessed by measuring the level of antioxidants and the concentration of substances derived from the action of oxygen free radicals on biological molecules, such as MDA. Our study demonstrated that a low dose of PET microplastics increased the level of MDA, indicating heightened oxidative stress. Concurrently, we observed a decrease in the activity of key antioxidant enzymes– CAT, SOD, GST. The reduced capacity of the antioxidant defense system plays an important role in the development of pancreatic β-cell dysfunction and is characteristic of early acute pancreatitis [53, 54]. In addition, the elevated MDA level indicates increased lipid peroxidation, reflecting oxidative damage to pancreatic cells by oxygen free radicals [55]. The fact that the low dose had only a minimal effect on the transcriptomic profile of the pancreas, while it had a significant effect on oxidative status, which suggests that both low and high doses of PET microplastics can affect pancreatic function, though different mechanisms. While the high dose may induce more pronounced transcriptional changes detectable by RNA-Seq, the low dose appears to induce oxidative stress through mechanisms that do not require extensive changes in gene expression or there are already changes at other molecular levels. Furthermore, the lack of correlation between gene expression and enzyme activity might be due to many factors, including differences in mRNA and protein stability or transcriptional and post-transcriptional regulation [56, 57]. In addition, functional feedback loops may also play a role, in which high concentrations of antioxidant enzymes suppress further gene expression or high mRNA levels do not necessarily lead to increased protein activity due to regulatory mechanisms [58].

In conclusion, our study shows that orally ingested PET microplastics disrupt pancreatic gene expression and oxidative balance in a dose-dependent manner. The upregulation of immune-related genes and the impairment of antioxidant defenses may indicate inflammation-induced damage to β-cells and a possible role in the development of diabetes. These results emphasize the biological effects of PET microplastics and the urgent need for further research into their contribution to metabolic diseases.

## Materials and methods

### Animals

All experimental protocols were approved by the Local Ethics Committee of the University of Warmia and Mazury in Olsztyn (Decision No. 10/2020, dated February 26, 2020). All methods were carried out in accordance with Polish law, which defines the conditions and methods of conducting experiments on animals, and the European Community Directive (EU Directive 2010/63/EU) on the ethical use of experimental animals. All methods were performed in accordance with ARRIVE guidelines. The animals, sourced from a farm in Lubawa, Poland, were kept under standard laboratory conditions with unrestricted access to fresh water (ad libitum) and a nutritionally appropriate diet. The housing environment was maintained at a temperature of 20–22 °C, with humidity levels controlled between 55–60%. To eliminate potential plastic contamination, all plastic materials were removed from the surroundings, and stainless steel was used for feeding and watering equipment. Prior to the experiment, the farm infrastructure where the gilts were housed also consisted of stainless steel. Bedding materials, including wood, were provided to ensure high animal welfare standards.

The study lasted four weeks and involved 15 eight-week-old gilts (Pietrain × Duroc) with an average body weight of approximately 20 kg. The animals were randomly divided into three groups: control group (CTR, *n* = 5) – received empty gelatin capsules orally, low-dose group (LD, *n* = 5) – administered a daily oral dose of 0.1 g/pig of PET microplastics (MPs) encapsulated in gelatin, and high-dose group (HD, *n* = 5) – given 1 g/pig of PET MPs per day in gelatin capsules. Capsules were provided one hour before the morning feeding. The PET microplastics used in the experiment were in the form of semi-crystalline polyethylene terephthalate (PET) powder, obtained from Goodfellow Cambridge Ltd., UK (Cat. No. ES306031/1). The physical properties of the plastic particles have been detailed in previous studies. In brief, the particle sizes ranged from 7.6 to 416.9 µm, with a predominant diameter of 158.5 µm. The particles exhibited diverse shapes, including spherical, fibrous, and irregular forms, with both sharp and rounded edges [59]. After the four-week exposure period, the animals were humanely euthanized. The euthanasia protocol included an initial intramuscular administration of atropine (0.05 mg/kg, Polfa, Poland), followed by xylazine (3 mg/kg, Vet-Agro, Poland) and ketamine (6 mg/kg, Vetoquinol Biowet, Poland). Approximately 20 min later, once full anesthesia was confirmed, an intravenous overdose of sodium pentobarbital (0.6 mL/kg, Biowet, Poland) was administered. The absence of vital functions was verified by the lack of a pupillary reflex, pulse, and respiration. Immediately after confirmation, pancreatic tissue samples were collected for subsequent analysis.

### RNA isolation, library preparation and sequencing procedure

RNA was isolated from 15 samples using the RNeasy Mini Kit (Qiagen, Germany) in accordance with the manufacturer’s guidelines. The quality and concentration of the extracted RNA were assessed using a Tecan Infinite M200 plate reader (Tecan Group Ltd., Switzerland), while RNA integrity was verified with an Agilent Bioanalyzer 2100 (Agilent Technologies, USA). The procedure for library preparation and sequencing has been previously detailed [51]. In summary, mRNA was first fragmented and then converted into cDNA using the TruSeq Stranded mRNA LT Sample Prep Kit (Illumina, San Diego, CA, USA). Unique adapters were attached to the double-stranded cDNA fragments, ensuring strand specificity. The prepared libraries were then pooled and sequenced on the Illumina NovaSeq 6000 platform using a paired-end (PE) 2 × 150 bp sequencing strategy.

### Quality control and genome mapping

The quality of raw paired-end reads was assessed using FastQC and Trimmomatic. Sequences were filtered based on the following criteria: (a) a minimum length of 120 bp, (b) a PHRED score greater than 20, and (c) uniform read length. High-quality trimmed reads were then aligned to the Sus scrofa 11.1 genome assembly using the STAR Aligner, referencing ENSEMBL annotation (release 98). The mapping results were subsequently indexed and sorted by genomic coordinates. Gene expression levels were obtained by processing the ballgown files and executing the prepDE.py script.

### Differentially expressed genes

The identification of differentially expressed genes (DEGs) was performed using DESeq2, applying a false discovery rate (FDR < 0.05) and a fold-change threshold of absolute log2 FC > 1.5. Changes in gene expression patterns in the porcine pancreas following in vivo exposure to PET microplastics were examined through high-throughput transcriptome sequencing. The transcriptomic effects of PET microplastics were evaluated across three comparisons: low-dose PET MPs (LD PET) versus control (CTR), high-dose PET MPs (HD PET) versus CTR, and HD PET versus LD PET. Additionally, fragments per kilobase of transcript per million mapped reads (FPKM) were computed as a normalized expression metric, considering sequencing depth and genomic feature length. Enrichment analysis of key biological processes and metabolic pathways among DEGs was conducted using the enrichGO and enrichKEGG functions from the clusterProfiler R package. The following parameters were used: Organism – pig, Ontology categories – CC, MF, or BP, *p*-adjusted value cut-off – 0.05, and p-adjustment method – BH. Visualization of expression and functional profiles was carried out using R Bioconductor packages such as ggplot2, circlize, and GOplot [60, 61].

### Real-time PCR validation

Differentially expressed genes were randomly selected for validation by real-time PCR using the AriaMx real-time PCR system (Agilent Technology, USA) as previously described [62]. Primer sequences (Supplemental Table S4) for the reference and target genes (*CCL22*, *CXCL10*, *IL12B*, *IL2R*, *TNFSF14, IRF8, JAK3)* were designed using Primer Express Software 3 (Applied Biosystems, USA). PCR reaction mixes with a final volume of 25 μl consisted of cDNA (4 ng), 300 μM of each primer, 12.5 μl of Power SYBR Green PCR Master Mix (Applied Biosystems, USA) and RNase-free water. The abundance of the tested mRNAs was calculated using the comparative Pfaffl method [63]. The constitutively expressed *PPIA* and *RPLP4* genes were implemented as reference genes, and the geometric mean values of the expression levels were used for analysis. Real-time PCR results were analyzed using Statistica software (version 13.1; Statsoft Inc. Tulsa, OK, USA) with Student’s t-test and expressed as means ± SEM. The results were considered statistically significant at *p* ≤ 0.05.

### Oxidative stress and antioxidant activity

Pancreatic tissue homogenates were prepared to analyze the oxidative status. Approximately 25 mg of the pancreatic tissue samples were homogenized according to the manufacturer’s instructions to ensure the integrity of the enzymatic activities. The homogenates were then subjected to various assays to determine oxidative stress marker and antioxidants activities using commercial kits from Cayman Chemical (USA) – Superoxide Dismutase Assay Kit (SOD, 706,002), Catalase Assay Kit (CAT, 707,002), Glutathione Peroxidase Assay Kit (GPx, 703,102), Glutathione S-Transferase Assay Kit (GST, 703,302), and the TBARS (TCA Method) Assay Kit (700,870) for the measurement of malondialdehyde (MDA). All enzymatic activities, with the exception of MDA, were normalized to the protein concentration determined using the BCA Protein Assay Kit (ThermoFisher, USA). Absorbance measurements for the assays were performed using the Infinite M200 Pro Reader in conjunction with Tecan i-control software (Tecan, Switzerland), following the manufacturer’s instructions for each assay kit. The results were analyzed using Statistica software (version 13.1; Statsoft Inc. Tulsa, OK, USA) with Student’s t-test and expressed as means ± SEM. The results were considered statistically significant at *p* ≤ 0.05.

## Supplementary Information

Supplementary Material 1.

## Data Availability

The raw data generated for this study can be found in the European Nucleotide Archive (ENA) with the accession number PRJEB80780.
