# Supplementary material for: Oral exposure to PET microplastics induces the pancreatic immune response and oxidative stress in immature pigs
Source: BMC Genomics. 2025 Jul 1;26:578. doi: 10.1186/s12864-025-11760-1 (PMC12211908; doi:10.1186/s12864-025-11760-1)
Supplement: Supplementary file 1 — Supplementary Material 1. [file 12864_2025_11760_MOESM1_ESM.zip › Supplemental data description.docx]

**Supplemental Fig. S 1**

Real-time PCR validation of RNA-seq results for random DEGs. Validation was performed for *CCL8*, *IL-1β*, *CCL4*, *OXTR* and *CSF3*.

**Supplemental Table S 2**

List of top 20 DEGs in the porcine pancreas after treatment with (A) high dose of PET microplastics and (B) comparison between high and low dose of PET microplastics.

**Supplemental Table S 2**

Genes identified in the porcine pancreas after treatment with (A) low dose of PET microplastics, (B) high dose, and (C) comparison between high and low dose of PET microplastics.

**Supplemental Table S 3**

Results of Gene Ontology enrichment analysis of DEGs significantly modulated in the porcine pancreas after treatment with (A) low dose of PET microplastics, (B) high dose, and (C) comparison between high and low dose of PET microplastics.

**Supplemental Table S 4**

Results of KEEG enrichment analysis of DEGs significantly modulated in the porcine pancreas after treatment with (A) low dose of PET microplastics, (B) high dose, and (C) comparison between high and low dose of PET microplastics.

**Supplemental Table S 5**

The primer sequences for the reference and target genes using in Real-time PCR validation.
