# Supplementary figures and images for: Oral exposure to PET microplastics induces the pancreatic immune response and oxidative stress in immature pigs
Source: BMC Genomics. 2025 Jul 1;26:578. doi: 10.1186/s12864-025-11760-1 (PMC12211908; doi:10.1186/s12864-025-11760-1)

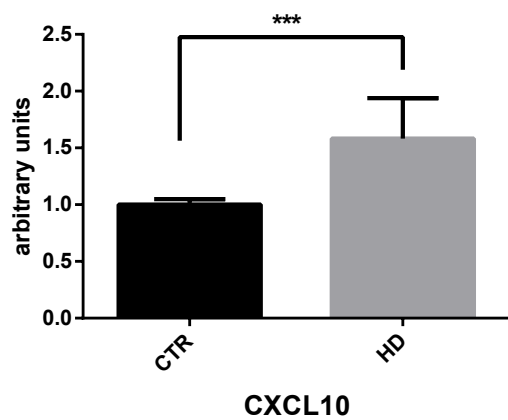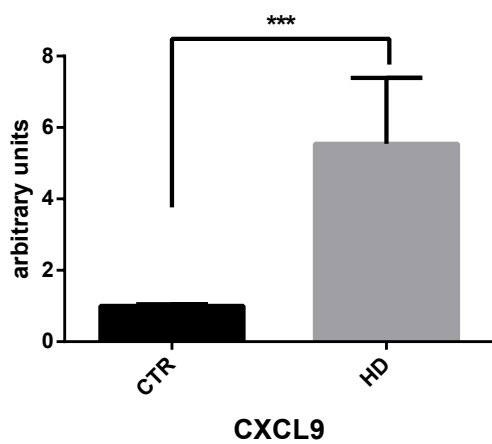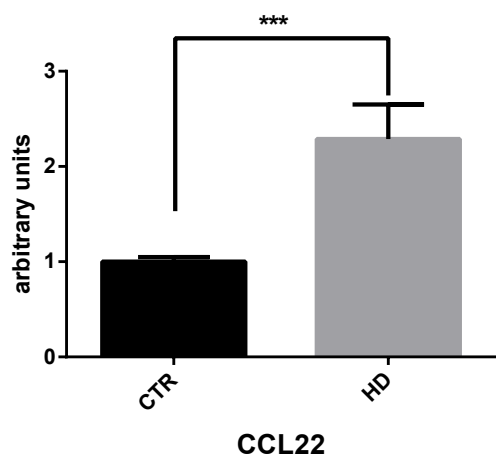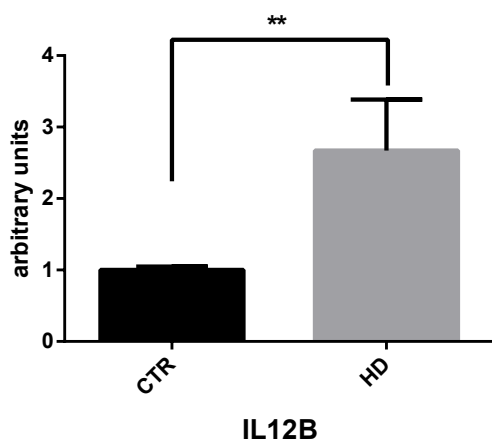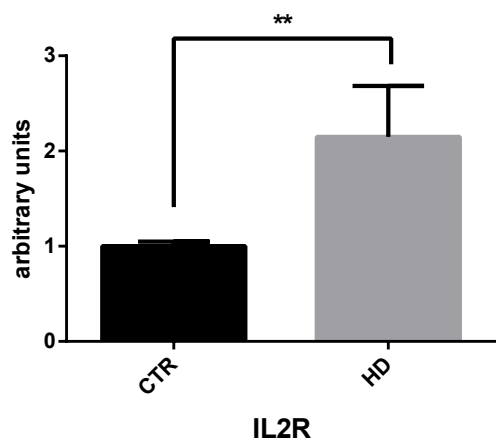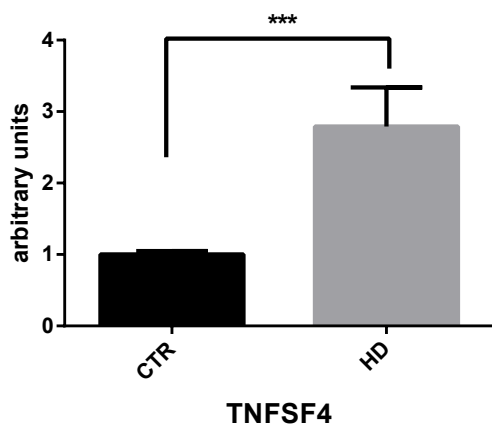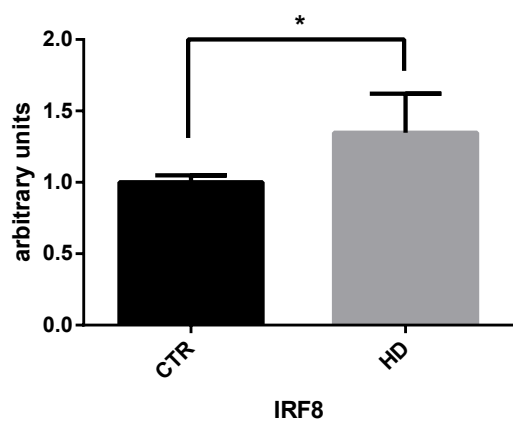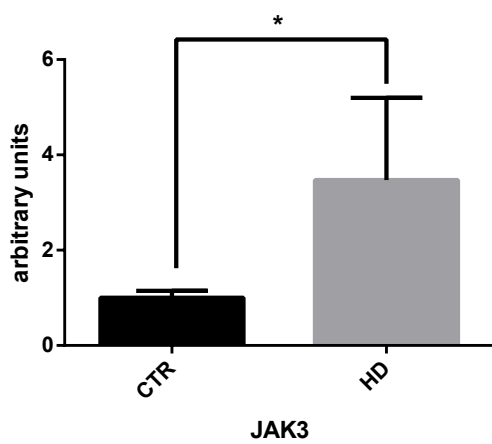

Supplement: Supplementary file 1 — Supplementary Material 1. [file 12864_2025_11760_MOESM1_ESM.zip › Supplemental Figure S1.pdf]
